# Distinct mechanisms of axonal globule formation in mice expressing human wild type α-synuclein or dementia with Lewy bodies-linked P123H ß-synuclein

**DOI:** 10.1186/1756-6606-5-34

**Published:** 2012-09-26

**Authors:** Akio Sekigawa, Masayo Fujita, Kazunari Sekiyama, Yoshiki Takamatsu, Taku Hatano, Edward Rockenstein, Albert R La Spada, Eliezer Masliah, Makoto Hashimoto

**Affiliations:** 1 Division of Sensory and Motor Systems, Tokyo Metropolitan Institute of Medical Science, Tokyo 156-8506, Japan; 2 Department of Neurology, Juntendo University School of Medicine, 2-1-1 Hongo, Tokyo, Bunkyo, 113-8421, Japan; 3 Department of Neurosciences, University of California-San Diego, La Jolla, CA, 92093-0624, USA; 4 Department of Pediatrics, and Cellular and Molecular Medicine, University of California-San Diego, La Jolla, CA, 92093-0624, USA; 5 Rady Children’s Hospital, San Diego, CA, 92193, USA

**Keywords:** α-synuclein, P123H β-synuclein, Parkinson’s disease, Mitochondria, Lysosome, Transgenic mouse

## Abstract

**Background:**

Axonopathy is critical in the early pathogenesis of neurodegenerative diseases, including Parkinson’s disease (PD) and dementia with Lewy bodies (DLB). Axonal swellings such as globules and spheroids are a distinct feature of axonopathy and our recent study showed that transgenic (tg) mice expressing DLB-linked P123H β-synuclein (P123H βS) were characterized by P123H βS-immunoreactive axonal swellings (P123H βS-globules). Therefore, the objectives of this study were to evaluate α-synuclein (αS)-immunoreactive axonal swellings (αS-globules) in the brains of tg mice expressing human wild-type αS and to compare them with the globules in P123H βS tg mice.

**Results:**

In αS tg mice, αS-globules were formed in an age-dependent manner in various brain regions, including the thalamus and basal ganglia. These globules were composed of autophagosome-like membranous structures and were reminiscent of P123H βS-globules in P123H βS tg mice. In the αS-globules, frequent clustering and deformation of mitochondria were observed. These changes were associated with oxidative stress, based on staining of nitrated αS and 4-hydroxy-2-nonenal (4-HNE). In accord with the absence of mitochondria in the P123H βS-globules, staining of nitrated αS and 4-HNE in these globules was weaker than that for αS-globules. Leucine-rich repeat kinase 2 (LRRK2), the PARK8 of familial PD, was detected exclusively in αS-globules, suggesting a specific role of this molecule in these globules.

**Conclusions:**

Lysosomal pathology was similarly observed for both αS- and P123H βS-globules, while oxidative stress was associated with the αS-globules, and to a lesser extent with the P123H βS-globules. Other pathologies, such as mitochondrial alteration and LRRK2 accumulation, were exclusively detected for αS-globules. Collectively, both αS- and P123H βS-globules were formed through similar but distinct pathogenic mechanisms. Our findings suggest that synuclein family members might contribute to diverse axonal pathologies.

## Background

α-Synucleinopathies such as Parkinson's disease (PD) and dementia with Lewy bodies (DLB) are leading causes of movement disorders and dementia in aging populations [1,2]. α-Synucleinopathies are characterized by the presence of Lewy bodies and Lewy neurites, which are filled with aggregates of α-synuclein (αS), an abundant nerve terminal protein with unknown functions. It is well established that αS has a central role in the pathogenesis of these diseases, but little is known about the onset and progression of the degenerative process.

Recently, evidence has accumulated to indicate that an axonal pathology caused by αS may play a critical role in the early pathogenesis of α-synucleinopathies. This is supported by the widespread axonal pathology observed from the earliest stages of these disorders, suggesting that axonal function may be impaired in the early pathogenesis [3]. In this context, the appearance of αS-positive Lewy neurites has been shown to precede that of Lewy bodies in brains and cardiac sympathetic neurons. These results suggest that degeneration begins in the distal axon and proceeds towards the cell body in α-synucleinopathies [4,5]. Thus, elucidation of the mechanisms of axonal pathology is important to gain a better understanding of the early pathogenesis of α-synucleinopathies and to establish effective therapeutic agents.

Axonal pathologies such as axonal deposits of αS and axonal swellings have been shown in various lines of transgenic (tg) mice expressing either wild-type αS or βS with PD-linked missense mutations [6-9], but have not been characterized extensively. Furthermore, not only αS, but also two αS-related molecules, β-synuclein (βS) and γ-synuclein (γS), are associated with neuritic pathology [10,11], such as that in dystrophic neurites and spheroid structures, in the brain in synucleinopathies. Thus, it is unclear how the synuclein family of peptides is involved in the axonal pathogenesis. Based on our findings for formation of axonal swellings in tg mice expressing DLB-linked P123H βS [12], we wondered if these swellings might be a useful model to investigate the axonal pathology caused by each synuclein protein. In this context, the objective of the present study was to characterize axonal swellings of tg mice expressing human αS and to compare them with those found in P123HβS tg mice. The results suggest that axonal swellings found in these two types of mice may be formed by similar but distinct mechanisms.

## Results

### Age-dependent formation of αS-accumulated axonal swellings (αS-globules) in brains of αS tg mice

**Figure 1:**
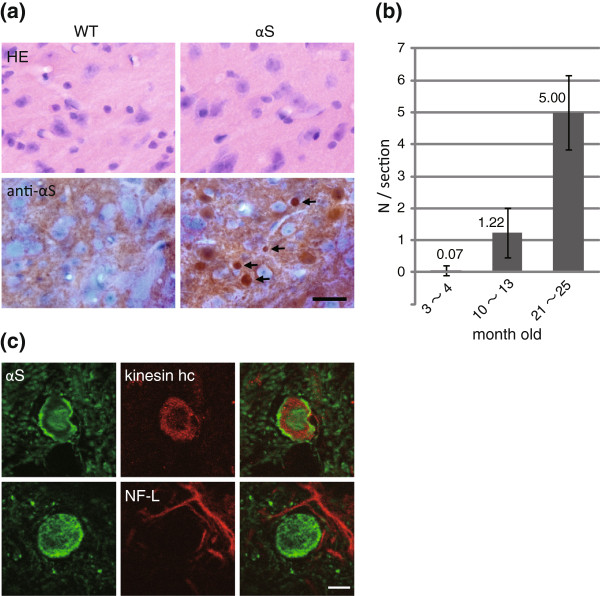
**Age-dependent formation of αS-immunopositive axonal swellings (αS-globules) in brains of αS tg mice.** (**a**) Hematoxylin and eosin staining (upper two panels) showed no apparent changes in brains of αS tg mice (right) and non-tg littermates (left). Immunohistochemistry of αS (lower two panels) showed formation of αS-globules (arrows) in αS tg mice (right), but not in wild type littermates (left). Representative images for the thalamus are shown. Scale bar = 20 μm. (**b**) Age-dependent formation of αS-globules in the αS tg brain detected by anti-αS antibody. The numbers of globules with diameters >4 μm were unbiasedly counted in the striatum of αS tg brains at three ages: young (n = 5, 3–4 mo), adult (n = 3, 10–13 mo), and old (n = 3, 21–25 mo). (**c**) Double immunofluorescence was performed using αS as a globule identification. Kinesin heavy chain (kinesin hc) was positive (upper three panels) and neurofilament-light (NF-L) was negative (lower three panels). Representative images are shown for the pontine nuclei (upper) and striatum (lower). Scale bar = 5 μm.

To evaluate αS-induced axonal pathologies in the brains of αS tg mice, various histological analyses were carried out. Hematoxylin and eosin staining showed no apparent changes (Figure 1a), but immunohistochemistry of brain sections of αS tg mice, but not of their wild type littermates, exhibited formation of strongly αS-immunoreactive axonal swellings in various areas, including the basal ganglia, thalamus, midbrain and olfactory bulb, but not in the cortex and cerebellum (Figure 1, Additional file 1: Figure S1a). These swellings formed in an age-dependent manner, with the highest number occurring in old stage (Figure 1b). The αS-immunoreactive swellings were occasionally immunopositive for heavy chain of kinesin as an axonal marker, but were not stained by eosin or anti-neurofilament-L antibody (Figure 1c), suggesting that they were a type of axonal swellings. In addition, the long-axis diameter of αS-immunoreactive swellings was 6.55 ± 2.56 μm (mean **±** S.D., n = 30 globules). Because the diameters of the swellings were less than 20 μm, they were categorized as “globules” (small spheroids). The swellings were not stained by Thioflavin T or Thiazine Red (data not shown) [13,14], suggesting that fibrillation of accumulated αS was not required for formation of αS-globules in brains of αS tg mice.

The αS-globules were immunopositive for several GABAergic markers, including anti-γ-aminobutyric acid (GABA) and anti-glutamic acid decarboxylase (Additional file 2: Figure S2a), and negative for other neuronal markers such as vesicular glutamate transporter-1 or −2, dopamine transporter, vesicular acetylcholine transporter and serotonin (data not shown). These results suggest that the αS-globules might be derived from GABAergic neurons. Furthermore, the αS-globules were highly immunopositive for calbindin D-28 k, but were partially positive for calretinin, and only occasionally positive for parvalbumin (Additional file 2: Figure S2b), suggesting that the globules might be derived from several types of GABAergic neurons. The mechanism through which globules caused by αS are preferentially formed in GABAergic neurons is unclear. However, our results are consistent with previous studies showing that both dopaminergic neurons and other neuronal types, including large cholinergic interneurons and medium-sized GABAergic projection neurons, are involved in the neuritic pathology in the neostriatum of the PD brain.

### Lysosomal pathology of αS-globules in brains of αS tg mice

**Figure 2:**
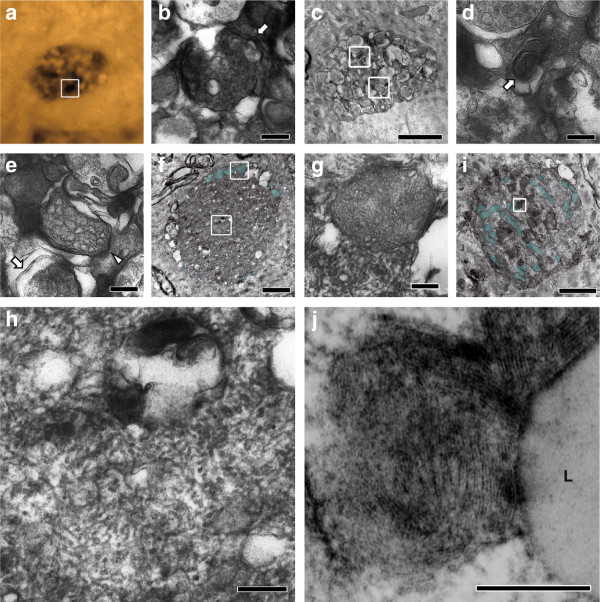
**Ultrastructure of αS-globules in brains of αS tg mice.** Immunoelectron microscopic analysis was performed using anti-αS. αS-immunopositive globules (**a**) were characterized by lysosomal pathologies such as an αS-immunopositive multivesicular body (**b**: arrow), autophagic vacuoles (**c**), myelinosome (**d**: arrow), a myelinoid membrane (**e**: arrow), and a light multivesicular body (**e**: arrow head). Formation of a fingerprint profile (**j**) adjacent to a lipid droplet (**j**: **L**) and curvilinear bodies (**f**, **h**) are reminiscent of lysosome storage disease. Accumulation of mitochondria was also occasionally observed (**f**, **i**; blue). Some mitochondria were swollen and deformed (**g**). The globules are representatives of those in the thalamus (**a**-**h**) and striatum (**i**, **j**). The boxed area in panels with lower magnifications (**a**, **c**, **f**, and **i**) were enlarged in **b**, and **j** or in two figures (**d**, **e**, **g**, **h**). Scale bar = 2 μm for **c**, **f**, **i**; 200 nm for **b**, **d**, **e**, **g**, **h**, **j**.

To investigate the ultrastructure of αS-globules in brains of αS tg mice, immunoelectron microscopy was performed (Figure 2). Similarly to the globules in P123H βS tg mice [15], the αS-globules in αS tg mice were characterized by membranous elements including autophagosome-like structures with double membranes (Figure 2a-e), multivesicular bodies (Figure 2b, e) and multilayered membranes (Figure 2d). These results suggest a possible relevance to aberrant regulation of the autophagy-lysosomal system. Notably, unique membranous structures, comprised of alternating dense and light band forms with a periodicity of 5.8-6.1 nm units and an electron-dense line thickness of 3.3-3.6 nm units, were present in the αS-globules (Figure 2i, j). Moreover, the tubular inclusions (13- to 18-nm diameter) existed in the αS-globules (Figure 2f, h). These structures were reminiscent of the fingerprint profile [16] and curvilinear body [17] that are frequently associated with lysosomal storage diseases such as neuronal ceroid-lipofuscinosis and gangliosidosis. Neither such membranous structures nor Lewy body-like filamentous structures were observed in the somata of αS-expressing neurons.

To characterize the lysosomal pathology in the αS-globules in more detail, an immunofluorescence study was carried out. The globules were immunopositive not only for major gangliosides (GD1a and GM1) but also for some minor gangliosides (GD3, GM2 and GM3) [18,19] (Additional file 3: Figure S3). Based on our previous reports regarding the protective effects of gangliosides on lysosomal pathology in neuroblastoma cells expressing P123H ßS, we speculate that gangliosides might be protective against formation of globules. Finally, the activities of cathepsins B and -D were significantly decreased in αS tg mice compared with non-tg littermates (The mean activity of cathepsin B was 69.8 ± 14.2% and that of cathepsin D was 86.6 ± 10.6%) (Additional file 1: Figure S1, Additional file 4: Additional Methods). These results suggest, but do not prove, that autophagosome-like membranes might accumulate due to decreased clearance by lysosomes. Essentially similar results were previously observed in brains of P123H ßS tg mice [15].

### Enhanced oxidative stress with mitochondrial abnormality in αS-globules in brains of αS tg mice

Besides a lysosomal pathology, immunoelectron microscopy showed accumulation of mitochondria in αS-globules in brains of αS tg mice. Some αS-globules displayed clustering of mitochondria (Figure 2f, i), while others had swollen mitochondria in the peripheral regions of the globules (Figure 2g). Consistent with the deformation of the mitochondria, there was a clear decrease in their osmophility (Figure 2g), indicating increased pH in the intermembrane space of mitochondria. However, more severe mitochondrial pathologies, such as distorted and vacuolated mitochondria, were not observed.

**Figure 3:**
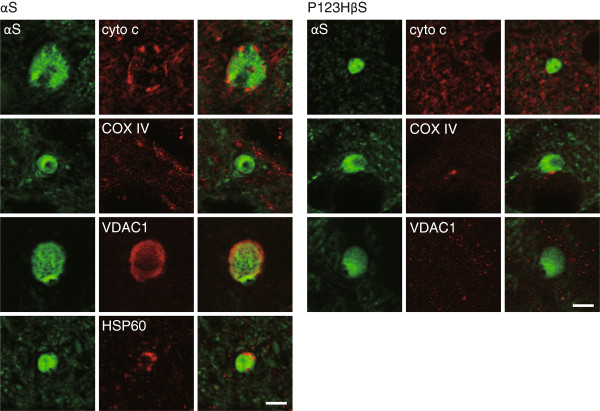
**Localization of mitochondrial marker proteins in αS-globules and P123H ßS-globules.** Because P123H βS-immunopositive globules in brains of P123H βS tg mice were immunopositive for αS (~100%), double immunofluorescence analyses of αS tg mice (twelve left panels) and P123H ßS tg mice (nine right panels) were performed using αS as a globule identification. In αS tg mice, cytochrome C (upper panels) and HSP60 (lowest panels) showed punctate patterns, while VDAC1 (third panels) was located diffusely. In contrast, COX IV (second panels) was not detected. In P123H βS tg mice, cytochrome C (upper panels), COX IV (middle panels) and VDAC1 (lower panels) were all immunonegative. Representative images are shown for the thalamus in αS tg mice and the basal ganglia in P123H βS tg mice. Scale bar = 5 μm.

To characterize the mitochondrial pathology in the αS-globules, an immunofluorescence study was conducted. The results showed that αS-globules were immunopositive for various mitochondria markers, including voltage-dependent anion channel isoform 1 (VDAC1), cytochrome C and the stress protein heat shock protein 60 (HSP 60) (Figure 3). All VDAC1 immunohistochemical images in αS-globules showed a diffuse pattern (67% in the thalamus, n = 12). This pattern of VDAC1 staining suggested possible damage of mitochondrial outer membrane [20]. However, it is unlikely that apoptosis was involved since cytochrome C and HSP 60 staining was still localized in the swollen mitochondria. The absence of COX IV, a cytochrome C oxidase subunit in the mitochondrial inner membrane, in the αS-globules is consistent with a report showing that genes derived from mitochondrial DNA, including COX IV, are deleted in many cases of sporadic PD [21].

**Figure 4:**
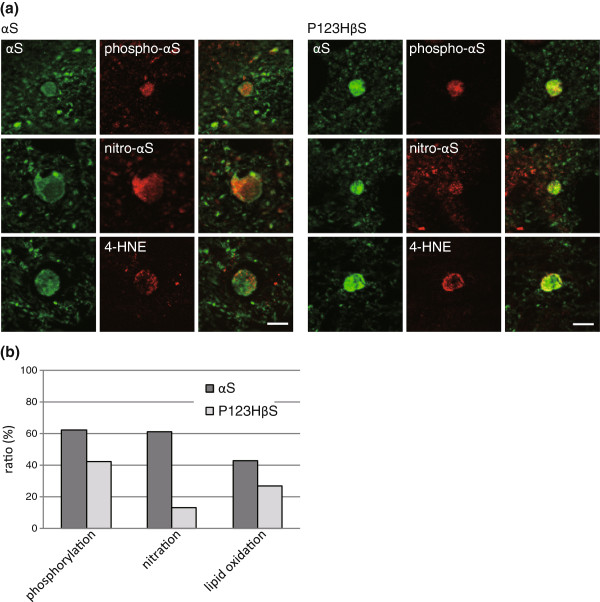
**Characterization of αS modification and oxidative stress for αS-globules and P123H ßS-globules.** (**a**) Double immunofluorescence analyses of αS tg mice (nine left panels) and P123H ßS tg mice (nine right panels) were performed using αS as a globule identification. Phosphorylation of αS, nitration of αS, and 4-HNE staining occurred to different extents in the two types of mice. Representative images are shown for the basal ganglia and thalamus in αS tg mice and for the basal ganglia in P123H βS tg mice. Scale bar = 5 μm. (**b**) Quantification of data for phosphorylation of αS, nitration of αS, and 4-HNE in the basal ganglia. (n = 3-4, over 18 mo).

Abnormal accumulation of mitochondria in αS-globules might stimulate oxidative stress. This possibility was assessed based on immunoreactivities to nitrated αS and 4-hydroxy-2-nonenal (4-HNE), a product of biological lipid peroxidation (Figure 4) [22,23]. In support of this hypothesis, considerable amounts of the αS-globules were immunostained with anti-nitrated-αS antibody (~61% in the basal ganglia, n = 59), suggesting that nitration was upregulated (Figure 4). Similarly, the αS-globules had the immunoreactivity for anti-4-HNE antibody (~43% in the basal ganglia, n = 54), confirmed that the oxidative stress was increased in the αS-globules (Figure 4). Phosphorylation of αS was evaluated as another possible posttranslational modification, since Lewy bodies in human brains are consistently immunopositive with anti-phospho-αS antibody [22,23]. In αS tg mice, many but not all of the αS-globules were stained with anti-phospho-αS antibody (~62% in the basal ganglia, n = 63), indicating that phosphorylation of αS may not be critical for globule formation.

### Oxidative stress without mitochondria in P123H ßS-globules in brains of P123H ßS tg mice

In contrast to the αS-globules, our previous ultrastructural study showed that mitochondria were rarely observed in P123H ßS-globules in brains of P123H ßS tg mice [15]. Similarly to the results in αS tg mice, P123H ßS tg mice had P123H ßS-immunopositive swellings (P123H ßS-globules) derived from GABAergic projection neurons which were immunoreactive for calbindin D-28 k, but were negative for both calretinin and parvalbumin [15]. We observed that the long-axis diameter of P123H ßS-globules (5.70 ± 1.15 μm, mean ± S.D., n = 30 globules) was comparable to that of αS-globules (6.55 ± 2.56 μm. p = 0.10, Student's *t*-test). Staining for VDAC1, cytochrome C and COX IV was negative in P123H ßS-globules in brains of P123H ßS tg mice (Figure 3). In accord with the absence of mitochondria, oxidative stress, as assessed by anti-4HNE antibody, in P123H ßS-globules in P123H ßS tg mice was less than that in αS tg mice (~27% in the basal ganglia, n = 55) (Figure 4). In a similar fashion, nitration of endogenous mouse αS in P123H ßS-globules was negligible (~13% in the basal ganglia, n = 54) (Figure 4), while phosphorylation of endogenous mouse αS in P123H ßS-globules was similar to that in αS-globules in the basal ganglia of αS tg mice (~42% in the basal ganglia, n = 55) (Figure 4).

**Figure 5:**
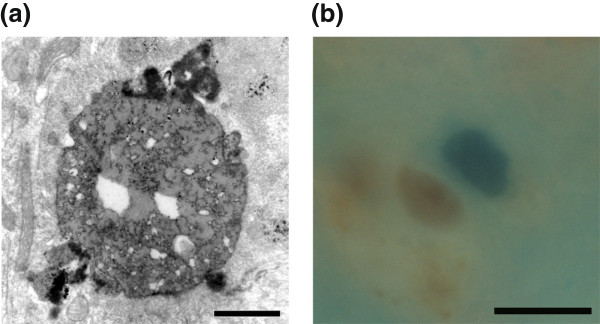
**Positive staining of cholesterol in P123H βS tg mice.** (**a**) An immunoelectron micrograph of αS-immunopositive globules in the basal ganglia of P123H βS tg mice showed many lipid droplets. Scale bar = 1 μm. (**b**) Detection of cholesterol in the basal ganglia of P123H βS tg mice was performed by Schultz staining. The arrowhead shows cholesterol-positive structure (blue), while the arrow indicates soma containing other lipids (brown). Scale bar = 10 μm.

The mechanism through which P123H βS stimulates formation of globules in the absence of mitochondria in axonal degeneration is unclear. We hypothesized that cholesterol might play a role in the pathogenesis, based on the results of immunoelectron microscopy for P123H ßS-globules in brains of P123H ßS tg mice, in which approximately half of the globules had accumulation of lipids droplets (Figure 5a). As we expected, cholesterol detection by Schultz staining was highly positive in globules in brains of P123H ßS tg mice (Figure 5b). In contrast, no staining of cholesterol was observed in globules in brains of αS tg mice.

### Analysis of familial PD-risk factors in globule formation

**Figure 6:**
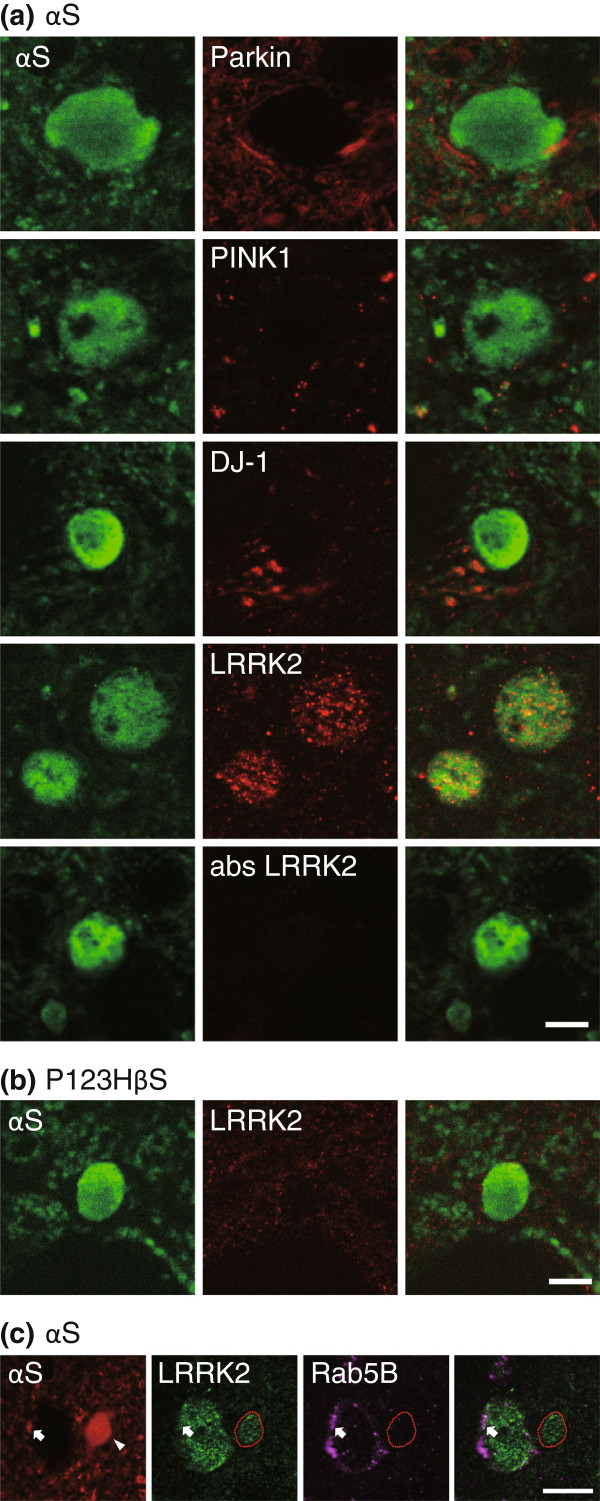
**LRRK2 accumulates in globules in αS tg mice.** (**a** and **b**) Double immunofluorescence for αS with parkin, PINK1, DJ-1, LRRK2, or negative control (the immunopeptide-preabsorbed anti-LRRK2 antibody) in αS tg mice (**a**) and P123H ßS tg mice (**b**). Note that αS-globules were immunopositive for LRRK2 (~79%, n = 22), whereas P123H ßS globules were negative for LRRK2. Representative images are shown for the thalamus (αS) and basal ganglia (P123H ßS). Scale bar = 5 μm for all panels. (**c**) Triple immunofluorescence for αS, LRRK2 and Rab5B for basal ganglia in αS tg mice. LRRK2 and Rab5B were colocalized in axon terminal (arrow), but were not colocalized in the αS-globule (arrowhead) Scale bar = 10 μm for all panels.

Since many familial PD risk factors [24,25] have been implicated in disorders of subcellular organelles such as lysosomes and mitochondria, we examined whether any of these factors were involved in globule formation in αS tg mice or P123H ßS tg mice. Notably, an immunofluorescence study showed frequent detection of leucine-rich repeat kinase 2 (LRRK2) (PARK8) in αS-globules (~79% in the thalamus, n = 28) (Figure 6a). The staining exhibited a small granular dot pattern, suggesting that LRRK2 might be associated with the membranous structures. The specificity of staining was confirmed by pre-absorption of the antibody with the immunogen peptides. In contrast, immunoreactivity of LRRK2 was not observed for P123H ßS-globules in the basal ganglia of P123H ßS tg mice (Figure 6b). It is intriguing if absence of LRRK2 in P123H ßS globule might reflect that LRRK2 strictly differentiates human αS from mouse αS and human P123H ßS. Alternative possibility to explain the differential expression of LRRK2 between the αS globule and P123H ßS globule is that LRRK2 might associate with some specific molecules which are expressed only in the αS globule. In this regard, rab5b could be such a candidate since a recent study has well characterized this molecule as a binding partner of LRRK2 [26]. Our immunofluorescence study showed that LRRK2 associates with an endosome molecule Rab5B in axon terminals with a normal range of size, but Rab5B was not detected in the αS-globules (Figure 6c). This result suggests a possibility that LRRK2 have lost the ability to interact with Rab5B, contributing to endosomal deficits during globule formation. Furthermore, although previous reports showed that LRRK2 associated various organelles, such as mitochondria and lysosome [26,27], we did not observed interaction of LRRK2 with mitochondria markers in the αS-globules (data not shown).

Furthermore, despite the accumulation of LRRK2 in the αS-globules, immunoblot analysis [28,29] failed to detect an apparent difference in LRRK2 bands among brain extracts derived from αS tg mice, P123H ßS tg mice, and their wild type littermates (data not shown), possibly due to the relatively small amount of LRRK2 in the αS-globules compared to total LRRK2 in the whole brain.

It has been well characterized that Parkin (PARK2) and PTEN-induced putative kinase 1 (PINK1) (PARK6) are autosomal recessive factors that are critically involved in the maintenance of mitochondrial quality, and that mutations in these genes are causative for mitophagy. However, neither Parkin nor PINK1 was immunopositive in αS- and P123H ßS-globules (Figure 6a). In addition, there was no immunoreactivity for DJ-1 (PARK7) in both types of globules (Figure 6a).

## Discussion

Axonal swellings, including globules and spheroids, are characteristic features of axonopathies observed in a number of diseases, including ischemia, trauma, neuroaxonal dystrophy, neurodegenerative disorders, as well as in aging. A recent study suggested that dysfunction of the autophagy-lysosome pathway could be one major contributor to axonal swellings [30,31]. Failure to degrade subcellular materials or organelles at distal axons and/or nerve terminals or failure to export these materials by axonal transport has been shown to produce swollen nerve terminals. Such a mechanism might be involved in formation of αS- and P123H βS-globules. In the present study, αS-globules in brains of αS tg mice were characterized by autophagosome-like membranous elements and were immunopositive for various minor gangliosides, which is reminiscent of some types of lysosomal storage disease. Consistent with this, lysosomal activity, as assessed by the activities of cathepsins B and -D, was significantly decreased in brain extracts of αS tg mice compared with those from non-tg littermates. Similar lysosomal dysfunctions were previously observed for P123H βS-globules in brains of P123H βS tg mice. Taken together, these results suggest that downregulation of the lysosome degradation pathway may be a common mechanism leading to globule formation in αS and P123H βS tg mice.

In contrast to the lysosomal pathology, mitochondria accumulated specifically in αS-globules. To the best of our knowledge, only one study has previously described abnormal mitochondria in the axonal pathology in tg mice expressing prion promoter-driven αS [32]. In agreement with this study, immunoelectron microscopy of αS revealed abnormal accumulation of mitochondria in αS-globules. Some αS-globules displayed clustering of mitochondria, while others had swollen mitochondria in the peripheral regions. Immunoreactivities of mitochondrial markers such as VDAC1 and cytochrome C were also found in αS-globules. These results suggest that mitochondria clustering might become hyperactive in response to lysosomal dysfunction. Consistent with these findings, αS-globules were associated with oxidative stress, as assessed by staining of 4-HNE and nitrated αS. Conversely, no evidence of mitochondria was obtained in P123H βS-globules, hence oxidative stress (assessed by 4-HNE staining) was less than that in αS-globules. The mechanism through which P123H βS causes mild level of oxidative stress without mitochondria is unclear, but it is noteworthy that cholesterol staining was positive in P123H βS-globules but not in αS-globules. Given that cholesterol and its metabolites are implicated in oxidative stress in the pathogenesis of neurodegenerative diseases [33], the increased oxidative stress in P123H βS-globules could be partly due to accumulation of cholesterol. A further study is warranted to test this intriguing possibility.

LRRK2 was found to be located in αS-globules and may be actively involved in the axonal pathology. Indeed, it was previously shown that LRRK2 was crucial for regulation of neurite formation and length. Knockdown of LRRK2 led to long, highly branched neuritic processes, whereas constructs with increased kinase activity exhibited short simple processes in neuronal cultures (or transduced nigrostriatal models) [34]. More recently, LRRK2R1441G BAC tg mice were shown to have various characteristic axonal pathologies, including large tyrosine hydroxylase-positive spheroid-like structures, dystrophic neurites and enlarged axonal endings [35]. Although the mechanisms are still unclear, the specific accumulation of LRRK2 in αS-globules naturally leads to the speculation that LRRK2 may cooperate with αS in the axonal pathology. In support of this possibility, both αS and LRRK2 have been shown to be commonly involved in pathologies such as impairment of cytoskeleton dynamics and dysregulation of the protein degradation system. Moreover, it was recently shown that various neuropathological features of A53T αS tg mice, such as impaired microtubule dynamics, Golgi disorganization, and decreased proteasomal activity, were worsened by cross-breeding with LRRK2 tg mice, but ameliorated by genetic ablation of LRRK2 [36]. Further investigation is required to determine whether αS and LRRK2 cooperate with each other to produce diverse pathologies, including axonal degeneration.

Finally, given that P123H βS may represent a rare familial case of DLB, it is important to consider whether wild type βS has any role in the formation of axonal globules in sporadic cases of α-synucleinopathies. In this context, neurite accumulation of βS has been demonstrated in various synucleinopathies, including PD, DLB, and neurodegeneration with brain iron accumulation, type I. Although wild type βS is neuroprotective, this molecule might become pathogenic during aging. It is also possible that wild type βS might become pathogenic under certain extreme conditions or through the action of specific environmental factors, leading to stimulation of globule formation. Thus, it is an intriguing possibility that the synuclein family of peptides might contribute to the formation of diverse axonal pathologies.

## Conclusions

The main objectives of this study were to evaluate αS-globules in the brains of tg mice expressing human wild-type αS and to compare them with the P123H βS-globules in P123H βS tg mice. The results showed lysosomal pathology was similarly observed for both αS- and P123H βS-globules. Oxidative stress was associated with the αS-globules, and to a lesser extent with the P123H βS-globules. Other pathologies, such as mitochondrial alteration and LRRK2 accumulation, were exclusively detected for αS-globules. Together, both αS- and P123H βS-globules were formed through similar but distinct pathogenic mechanisms, suggesting that synuclein family members might contribute to diverse axonal pathologies.

## Methods

All animal procedures were approved and conducted in accordance with the regulations of the Animal Ethics Review Committee of Tokyo Metropolitan Institute of Medical Sciences. *Thy1*-αS tg mice [37] and *Thy1*-P123H βS tg mice (line C) [15] were analyzed using various histological procedures.

### Histology and immunohistochemistry

#### Tissue preparation

The mice were anesthetized by overdose of pentobarbital and sacrificed by cardiac perfusion using 5 ml of an ice-cold solution of 250 mM sucrose and 5 mM MgCl_2_ in 0.02 M phosphate buffer (pH 7.4) (PB), followed by treatment with 4% paraformaldehyde, 15% saturated picric acid and 0.05% (for single or double-immunohistochemistry, and histochemistry), 0.5% (for immunoelectron microscopic analysis) or 1% (for GABA immunohistochemistry) glutaraldehyde in 0.1 M PB. Serial sections of 20- or 50-μm thickness were then prepared by a vibrating blade microtome (VT1200S; Leica, Nussloch, Germany). Tissue sections were put in glass tubes containing 15% sucrose in 0.1 M PB for 3 h, in 30% sucrose in 0.1 M PB for 3 h, and kept at −30°C until use [38].

#### Hematoxylin and eosin staining

Sections were stained with Mayer’s haematoxylin and 0.5% eosin. Sections were imaged using a Carl Zeiss (Jena, Germany) microscope.

#### Antisera

All antisera or monoclonal antibodies were purchased from commercial sources (Table 1).

**Table 1:** Primary and secondary antisera used in this study

	**Antigen**	**Host**	**Dilution**	**Mono/polyclonal**	**Source**
Primary antisera and antibodies					
1	α-synuclein	mouse	1:500 (for EM)	monoclonal	BD Biosciences (610787)
			1:1000 (for GABA FIHC)		
			1:2000		
2	α-synuclein	rabbit	1:50	polyclonal	Cell Signaling (#2628)
3	phosphorylated α-synuclein	mouse	1:2000	monoclonal	WAKO (pSyn#64)
4	nitrated α-synuclein	mouse	1:50	monoclonal	Santa Cruz Biotechnology (sc-32279)
5	4-hydroxy-2-nonenal	mouse	1:40	monoclonal	NOF Corporation (MHN-020P)
6	Calbindin D-28 k	rabbit	1:5000	polyclonal	Swant (CB38)
7	Parvalbumin	mouse	1:2000	monoclonal	Merck Millipore (MAB1572)
8	Calretinin	rabbit	1:2000	polyclonal	Swant (7699/4)
9	Glutamic acid decarboxylase	rabbit	1:2000	polyclonal	BIOMOL (GC3008)
10	GABA	rabbit	1:2000	polyclonal	Sigma (A2052)
11	kinesin, heavy chain	mouse	1:50	monoclonal	Merck Millipore (MAB1614)
12	neurofilament-ilght	mouse	1:200	monoclonal	Sigma (N5139)
13	GD1a	mouse	1:10	monoclonal	Seikagaku Corporation (GMR17)
14	GD3	mouse	1:10	monoclonal	Seikagaku Corporation (GMR19)
15	GM2	mouse	1:10	monoclonal	Seikagaku Corporation (GMB28)
16	GM3	mouse	1:10	monoclonal	Seikagaku Corporation (GMR6)
17	VDAC1	rabbit	1:40	polyclonal	Protein Tech (10866-1-AP)
18	cytochrome c	mouse	1:200	monoclonal	BD Biosciences (556432)
19	COX IV	rabbit	1:100	monoclonal	Cell Signaling (#4850)
20	HSP60	rabbit	1:50	polyclonal	Novus (NB100-91819)
21	Parkin	rabbit	1:100	polyclonal	Merck Millipore (AB9244)
22	PINK1	rabbit	1:50	polyclonal	Novus (NB600-973)
23	DJ-1	rabbit	1:50	polyclonal	abcam (ab74268)
24	LRRK2	rabbit	1:200	polyclonal	Novus (NB300-268)
25	VGAT	guinea pig	1:500	polyclonal	Synaptic Systems (131004)
26	Rab5B	goat	1:50	polyclonal	Santa Cruz Biotechnology (sc-26569)
Fluorescein conjugated probe					
1	Alexa 488 conjugated cholera toxin subunit B		0.5 μg/ml		invitrogen (C-34775)
Fluorescein conjugated secondary antisera					
1	Alexa 488 conjugated anti-mouse IgG	goat	1:200	polyclonal	invitrogen (A-11029)
2	Alexa 488 conjugated anti-rabbit IgG	goat	1:200	polyclonal	invitrogen (A-11034)
3	Alexa 594 conjugated anti-mouse IgG	goat	1:200	polyclonal	invitrogen (A-11032)
4	Alexa 594 conjugated anti-rabbit IgG	goat	1:200	polyclonal	invitrogen (A-11037)
5	Alexa 488 conjugated anti-mouse IgM	goat	1:100	polyclonal	invitrogen (A-21042)
6	Alexa 680 conjugated anti-goat IgG	donkey	1:100	polyclonal	invitrogen (A-21084)
Biotinylated secondary antisera					
1	biotinylated anti-mouse IgG	horse	1:200	polyclonal	Vector (BA-2000)

#### Immunohistochemistry

The sections were incubated in Tris-buffered saline (TBS) containing 1% sodium borohydrate for 30 min, in addition to treatment with TBS containing 1% H_2_O_2_ for 30 min in the case of diaminobenzidine staining. They were then incubated with primary antibodies (listed in Table 1) in PBS containing 1% normal horse serum and 0.4% Triton X-100 (except that for the lipids detection) overnight at 4°C, followed by detection with biotinylated secondary antibodies and an avidin-biotin complex kit (Vector Laboratories, Burlingame, CA) [39]. A positive reaction was detected using diaminobenzidine tetrahydrochloride (DAB) containing 0.01% hydrogen peroxide and counterstaining with hematoxylin. For detection with fluorescent dye, the sections were incubated with primary antibodies, followed by Alexa Fluor-conjugated secondary antibodies (Invitrogen, Carlsbad, CA). The sections were observed using a sectioning fluorescence microscopy system (Apotome; Carl Zeiss, Jena, Germany).

#### Immunoelectron microscopy

The sections were incubated in PB containing 1% sodium borohydrate for 30 min and in TBS containing 1% H_2_O_2_ for 30 min before incubation with primary antiserum against αS in TBS containing 10% normal goat serum and 2% bovine serum albumin overnight at 4°C. The sections were then incubated in biotin-conjugated secondary antiserum followed by treatment with ABC complex (Vector Laboratories) and staining with nickel-enhanced DAB. The stained sections were postfixed in 1% OsO_4_ in 0.1 M PB for 60 min, and then stained with 1% uranyl acetate and dehydrated in graded ethanol. Sections were flat embedded on silicon-coated glass slides in Quetol 812 (Nisshin EM, Tokyo, Japan). Immunopositive tissues were serially sectioned at 70-nm thickness with EM UC7 (Leica), followed by final staining with lead citrate. The labeled αS-globules were photographed using an H-7650 electron microscope (Hitachi, Tokyo, Japan) and image files were made from EM films using a scanner (GT-X970; Epson, Suwa, Japan) [38].

#### Cholesterol staining

The sections were incubated in 2.5% iron alum solution for 3 days at room temperature. Sections were onto slides, followed by draining the solution and drying. Schultz reagent (mixture of equal parts of glacial acetic acid and concentrated sulfuric acid) was applied onto the slide, and then a glass coverslip was mounted [40]. Sections were imaged using an Olympus (Tokyo, Japan) microscope.

### Globule counting

For the caudate and putamen, sagittal sections approximately 1.3-1.9 lateral to the midline were used. The location of the slice and identification of brain regions were determined by comparison to atlas images, as previously described [15]. Fluorescent labeled αS-immunopositive globules with a long axis ≥4 μm were counted directly under a fluorescent microscope or from photomicrographs of sections.

### Statistical analysis

Data are given as the means ± S.D. Statistical analysis was performed using SPSS (SPSS Inc. Chicago, IL). *T*-test was used for confirmation of significant differences among WT or P123HβS tg, and αS tg mice, with P < 0.05 considered to indicate a significant difference.

## Competing interests

The authors declare no competing financial interests.

## Authors’ contributions

AS, MF, KS, YT, and ER performed the experiments. AS, TH, ARL, EM and MH designed and analyzed the data. AS, ARL, EM and MH wrote the paper. All authors have read and approved the manuscript.

## Supplementary Material

Additional file 1 **Figure S1.** Lysosome and proteasome activities in the brain extracts of αS tg mice. (a) αS-globules were detected in the olfactory bulb (arrow), but not in the cerebellum, of old αS tg mice (24 mo). Scale bar=2 mm (upper panel), 50 μm (lower two panels). (b) Cathepsin B, -D and proteasome activities were measured ( Additional file 4: Additional Methods). Activities of lysosome (cathepsins B and –D) were significantly lower (p<0.05) in the olfactory bulb but not in the cerebellum in αS tg mice compared to the same areas in non-tg littermates (over 23 mo). In contrast, there were no significant difference in proteasome activities (Peptidyl-glutamyl peptide-hydrolyzing (PGPH) enzyme and chymotrypsin) between αS tg mice and non-tg littermates (mean±S.D.; *p<0.05, n=6 per group). Click here for file

Additional file 2 **Figure S2.** αS-globules are derived from GABAergic neurons. (a) αS-immunopositive globules in the striatum and thalamus of old αS tg mice (over 18 mo) were consistently immunopositive for GABA and glutamic acid decarboxylase (GAD), and were weakly immunopositive for vesicular GABA transporter (VGAT) (arrowhead). Scale bar=5 μm. (b) Immunoreactivity for calbindin (CB) was consistently observed. Staining was partially positive for parvalbumin (PV) and rarely positive for calretinin (CR) in the thalamus. Scale bar=5 μm. Click here for file

Additional file 3 **Figure S3.** Immunoreactivities of gangliosides in αS-globules of αS tg mice. (a) Double immunofluorescence analysis of αS tg mice was performed using αS as a globule identification. αS-immunopositive globules in the thalamus of old αS tg mice (25 mo) were positively stained with various anti-ganglioside antibodies. Scale bar=5 μm. (b) Quantification of these data. Click here for file

Additional file 4 **Additional Methods.** Measurement of lysosome and proteasome activity. Click here for file
