# Supplementary figures and images for: Distinct mechanisms of axonal globule formation in mice expressing human wild type α-synuclein or dementia with Lewy bodies-linked P123H ß-synuclein
Source: Mol Brain. 2012 Sep 26;5:34. doi: 10.1186/1756-6606-5-34 (PMC3546907; doi:10.1186/1756-6606-5-34)

(a)

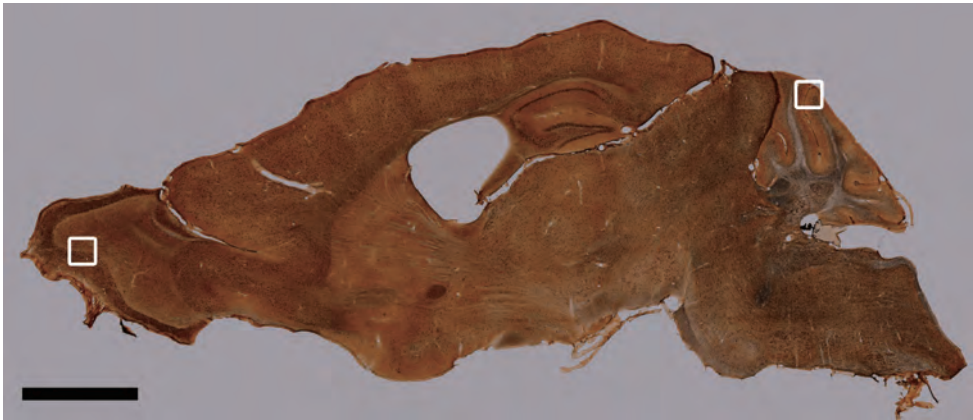

olfactory bulb

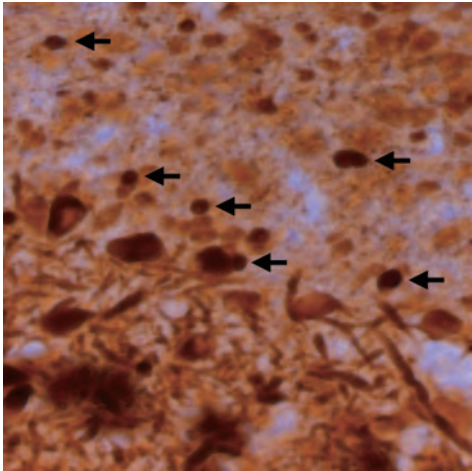

cerebellum

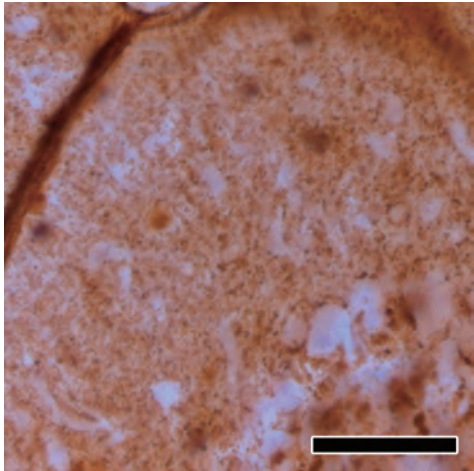

(b)

olfactory bulb

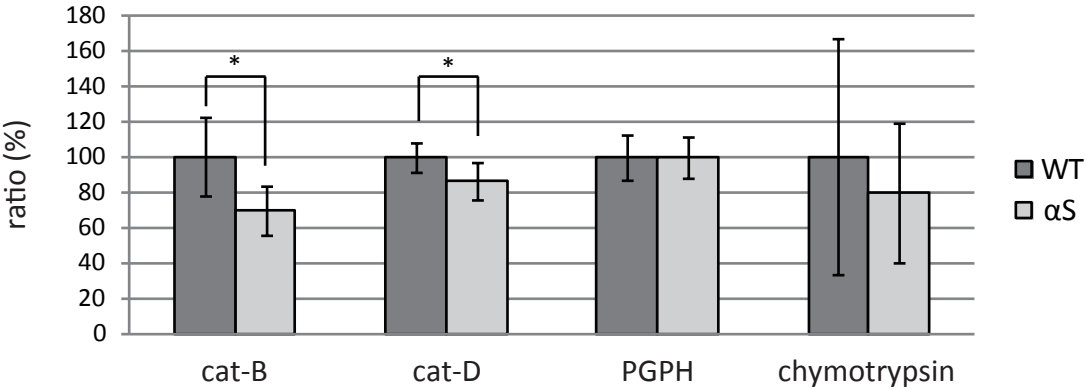

cerebellum

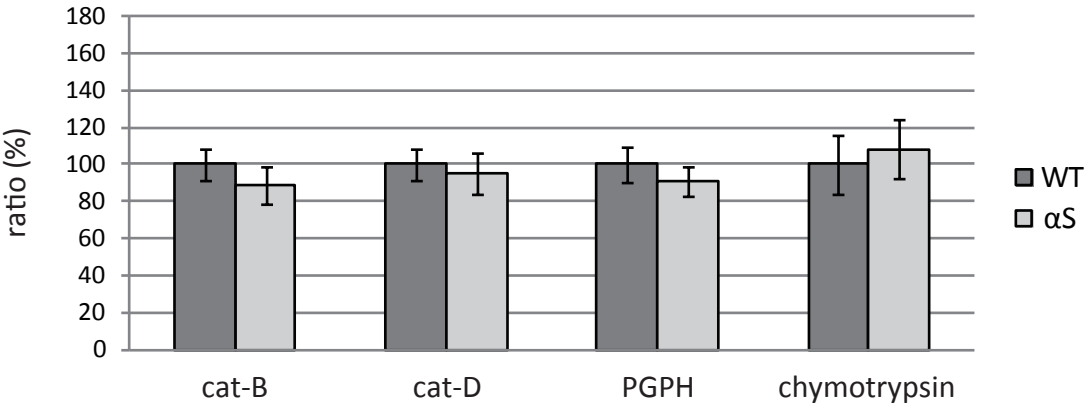

Supplement: Additional file 1 — Figure S1. Lysosome and proteasome activities in the brain extracts of αS tg mice. (a) αS-globules were detected in the olfactory bulb (arrow), but not in the cerebellum, of old αS tg mice (24 mo). Scale bar=2 mm (upper panel), 50 μm (lower two panels). (b) Cathepsin B, -D and proteasome activities were measured ( Additional file 4: Additional Methods). Activities of lysosome (cathepsins B and –D) were significantly lower (p<0.05) in the olfactory bulb but not in the cerebellum in αS tg mice compared to the same areas in non-tg littermates (over 23 mo). In contrast, there were no significant difference in proteasome activities (Peptidyl-glutamyl peptide-hydrolyzing (PGPH) enzyme and chymotrypsin) between αS tg mice and non-tg littermates (mean±S.D.; *p<0.05, n=6 per group). [file 1756-6606-5-34-S1.pdf]

(a)

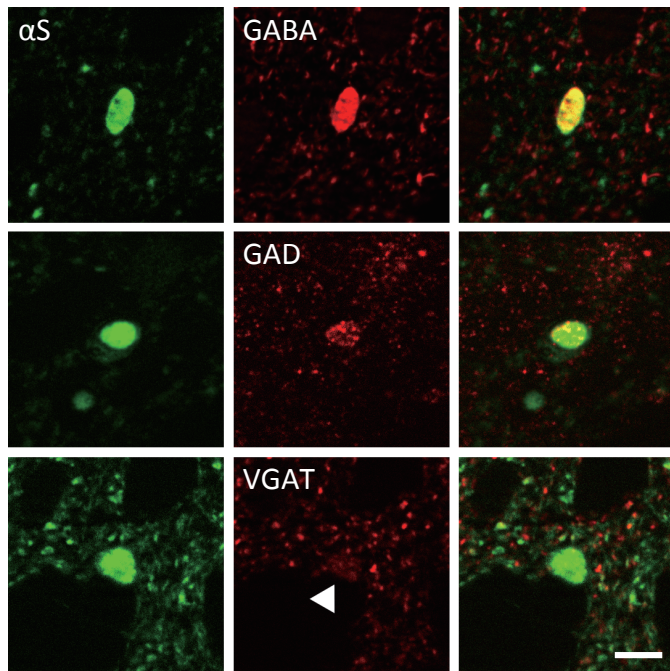

(b)

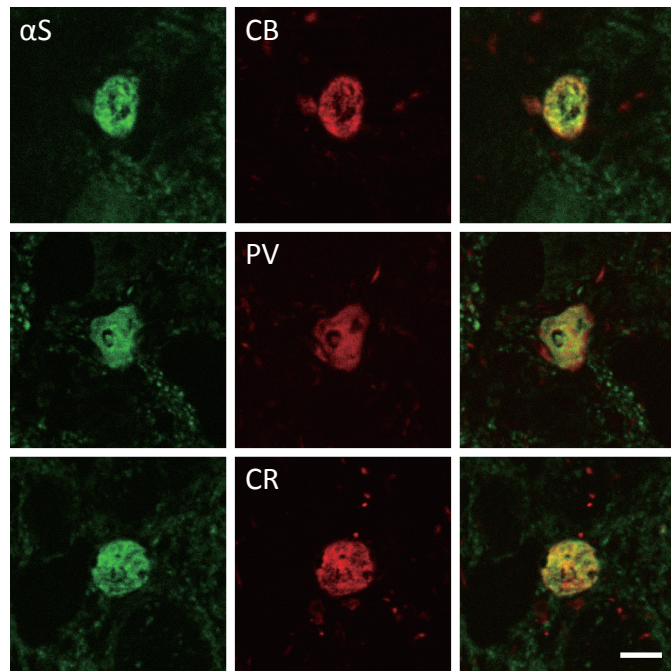

Supplement: Additional file 2 — Figure S2. αS-globules are derived from GABAergic neurons. (a) αS-immunopositive globules in the striatum and thalamus of old αS tg mice (over 18 mo) were consistently immunopositive for GABA and glutamic acid decarboxylase (GAD), and were weakly immunopositive for vesicular GABA transporter (VGAT) (arrowhead). Scale bar=5 μm. (b) Immunoreactivity for calbindin (CB) was consistently observed. Staining was partially positive for parvalbumin (PV) and rarely positive for calretinin (CR) in the thalamus. Scale bar=5 μm. [file 1756-6606-5-34-S2.pdf]

(a)

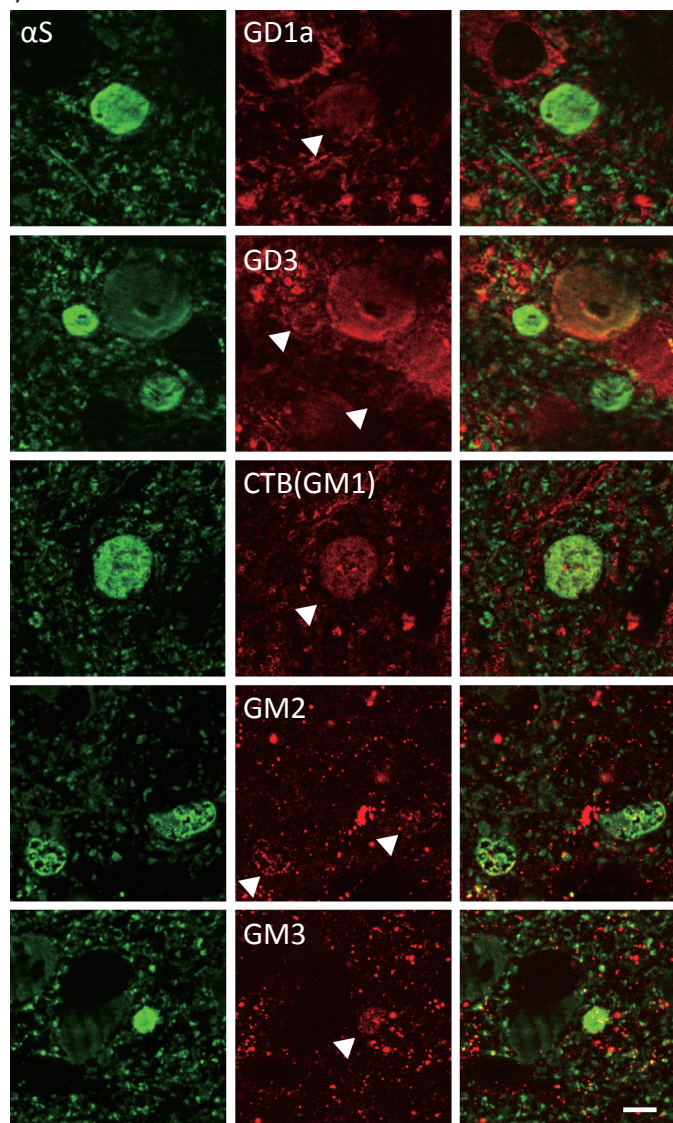

(b)

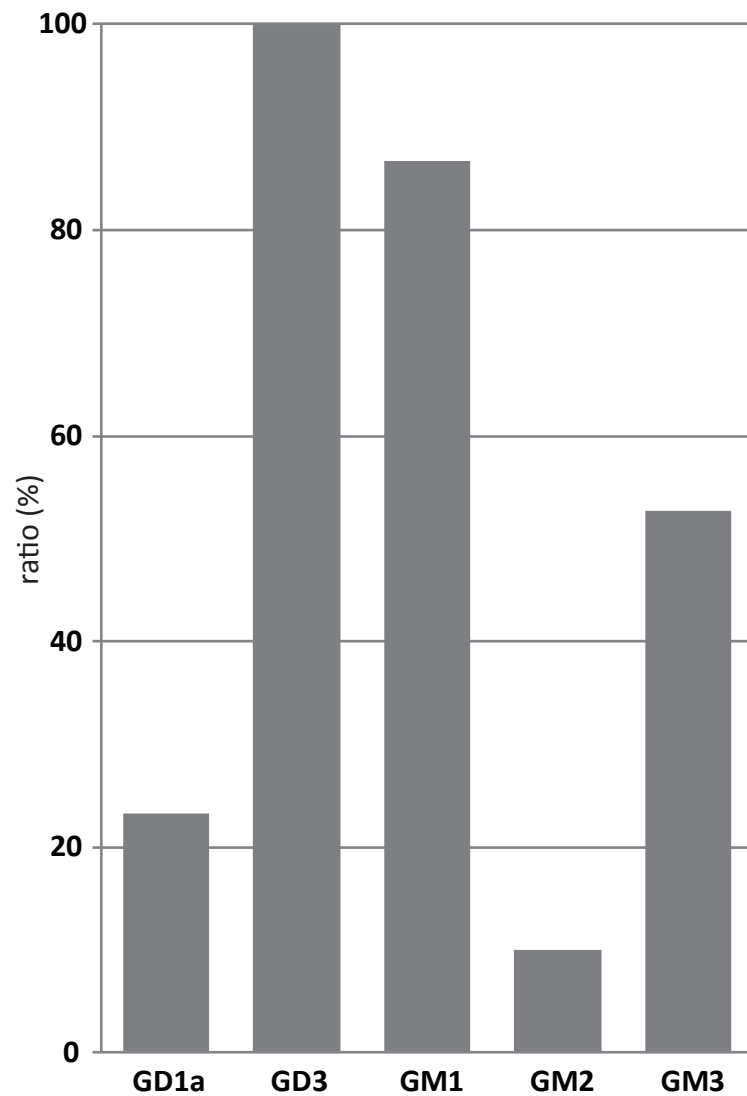

Supplement: Additional file 3 — Figure S3. Immunoreactivities of gangliosides in αS-globules of αS tg mice. (a) Double immunofluorescence analysis of αS tg mice was performed using αS as a globule identification. αS-immunopositive globules in the thalamus of old αS tg mice (25 mo) were positively stained with various anti-ganglioside antibodies. Scale bar=5 μm. (b) Quantification of these data. [file 1756-6606-5-34-S3.pdf]
