# Supplementary material for: Distinct mechanisms of axonal globule formation in mice expressing human wild type α-synuclein or dementia with Lewy bodies-linked P123H ß-synuclein
Source: Mol Brain. 2012 Sep 26;5:34. doi: 10.1186/1756-6606-5-34 (PMC3546907; doi:10.1186/1756-6606-5-34)
Supplement: Additional file 4 — Additional Methods. Measurement of lysosome and proteasome activity. [file 1756-6606-5-34-S4.pdf]

Additional Methods

Measurement of lysosome and proteasome activity.

Cathepsin B, -D and proteasome activities were measured as previously described[15]. Briefly, olfactory bulb and cerebellum extracts were constituted in buffer containing 50 mM HEPES (pH 6.0), 10 mM EDTA, and 10 mM NaCl, subjected to sonication to rupture cell membranous structures, and centrifuged at 100,000 × g for 30 min. Supernatants (10 μg) were incubated with either Z-RR-AMC fluorogenic cathepsin B substrate (40 μM) or Bz-RGFFP-4-MeObNA cathpsin D substrate (40 μM) (both from Chemicon, Temecula, CA). For measurement of proteasome activities, 10 μg of the supernatants were incubated in the extraction buffer (50 mM HEPES (pH 7.4), 10 mM EDTA, 10 mM NaCl) containing either Z-LLE-AMC fluorogenic substrate (40 μM) for peptidylglutamyl-peptide hydrolyzing (PGPH) activity or Z-VKM-AMC substrate (40 μM) (both from Chemicon) for chymotrypsin-like activity. The enzymatic activities were assayed by continuous recording of the fluorescence activity released from the fluorogenic substrate using a Berthold Mithras LB940 microplate reader (Berthold, Bad Wildbad, Germany) for 1 h at 37°C (excitation 380 nm, emission 460 nm for cathepsin B and proteasome and excitation 345 nm, emission 425 nm for cathepsin D), and the reaction rates were analyzed. The activities are given in arbitrary units/min/mg of protein and normalized to wild type activity.

Additional Figures

Additional Figure 1. αS-globules are derived from GABAergic neurons

(a) αS-immunopositive globules in the striatum and thalamus of old αS tg mice (over 18 mo) were consistently immunopositive for GABA and glutamic acid decarboxylase (GAD), and were weakly immunopositive for vesicular GABA transporter (VGAT) (arrowhead). Scale bar=5 µm. (b) Immunoreactivity for calbindin (CB) was consistently observed. Staining was partially positive for parvalbumin (PV) and rarely positive for calretinin (CR) in the thalamus. Scale bar=5 µm.

Additional Figure 2. Immunoreactivities of gangliosides in αS-globules of αS tg mice

(a) Double immunofluorescence analysis of αS tg mice was performed using αS as a globule identification. αS-immunopositive globules in the thalamus of old αS tg mice (25 mo) were positively stained with various anti-ganglioside antibodies. Scale bar=5 µm. (b) Quantification of these data.

Additional Figure 3. Lysosome and proteasome activities in the brain extracts of αS tg mice

(a) αS-globules were detected in the olfactory bulb (arrow), but not in the cerebellum, of old αS tg mice (24 mo). Scale bar=2 mm (upper panel), 50 µm (lower two panels). (b) Activities of lysosome (cathepsins B and –D) were significantly lower ($p<0.05$) in the olfactory bulb but not in the cerebellum in αS tg mice compared to the same areas in non-tg littermates (over 23 mo). In contrast, there were no significant difference in proteasome activities (Peptidyl-glutamyl peptide-hydrolyzing (PGPH) enzyme and chymotrypsin) between αS tg mice and non-tg littermates (mean±S.D.; *$p<0.05$, $n=6$ per group).
